# *Mycoplasma agassizii* Multilocus Sequence Typing Using Nanopore Sequencing: Insights Into Genetic Diversity and Isolate Characterization

**DOI:** 10.1111/nyas.70128

**Published:** 2025-10-30

**Authors:** Carla Cacciotto, Rosanna Zobba, Manuel Louro, Margarida Alves, Andreia Valença, Rui Patrício, Emanuela Bazzoni, Marco Pittau, Roberta Lecis, Alberto Alberti

**Affiliations:** ^1^ Department of Veterinary Medicine University of Sassari Sassari Italy; ^2^ Mediterranean Center for Disease Control Sassari Italy; ^3^ Faculty of Veterinary Medicine Lusófona University‐Lisbon University Center Lisbon Portugal; ^4^ I‐MVET ‐ Research in Veterinary Medicine, Faculty of Veterinary Medicine Lusófona University‐Lisbon University Center Lisbon Portugal; ^5^ CECAV ‐ Animal and Veterinary Research Center, Faculty of Veterinary Medicine Lusófona University‐Lisbon University Center Lisbon Portugal; ^6^ Superior School of Health, Protection and Animal Welfare Polytechnic Institute of Lusophony Lisbon Portugal; ^7^ All Pets – Clínica Veterinária de Tires Lisbon Portugal

**Keywords:** MLST, *Mycoplasma agassizii*, Nanopore sequencing, tortoises, URTD

## Abstract

*Mycoplasma agassizii* (*Mycoplasmopsis agassizii*) is a major pathogen responsible for upper respiratory tract disease (URTD) in tortoises, contributing to worldwide population declines. Despite its significance, tools for strain‐level identification and epidemiological tracking remain limited. This study proposes a Multilocus Sequence Typing (MLST) scheme based on eight housekeeping genes (*uvrB*, *tpiA*, *gyrB*, *efTu*, *rpoB*, *uvrA*, *gmK*, and *atpG*), combined with Nanopore sequencing, to characterize *M. agassizii* isolates from tortoises in Italy and Portugal. Among 127 samples, 73 (57.5%) tested positive for *M. agassizii*. Analysis of 15 isolates revealed 14 distinct sequence types (STs). High genetic diversity was observed, even among samples from the same rescue centers. No correlation was found between STs and geographic origin or tortoise species. Four loci (*uvrB*, *gyrB*, *rpoB*, and *gmK*) were sufficient to discriminate all STs at a haplotype diversity cut‐off ≥ 0.83. The standardized index of association (I_a_ = 0.2246) suggests a partially clonal population with evidence of recombination. This MLST scheme offers a promising tool for tracking *M. agassizii* strain diversity and understanding its epidemiology. Broader application across symptomatic and asymptomatic hosts is needed to evaluate associations between specific strains and disease outcomes, which will support conservation and URTD control efforts in tortoises.

## 1 Introduction

Tortoises have been an integral part of global ecosystems for about 220 million years, playing an important yet overlooked ecological role. Indeed, tortoises are involved in many ecological processes, such as seed dispersal, carbon storage, and mineral cycling, and are pivotal for the maintenance of global biodiversity and ecological balance [1]. However, tortoise populations have increasingly declined over the past years [2]. This decline in terrestrial tortoise populations is driven by a combination of human‐induced environmental changes and the emergence and spread of infectious agents [3]. Tortoises face a range of significant threats, including climate change, habitat encroachment and degradation, individuals and eggs harvesting for human consumption, illegal pet trade, and the production of turtle‐derived products in traditional medicine [3, 4]. Furthermore, the introduction of alien invasive plants, coupled with climate change, contributes to tortoise habitat loss by exacerbating wildfires frequency and intensity [5].

Tortoise populations are significantly threatened by infectious diseases caused by bacteria (such as mycoplasmas [6]) and viruses (e.g., herpesvirus [7] and ranavirus [8]). Among them, *Mycoplasma agassizii* (*M. agassizii*) is a major concern for tortoises, being associated with upper respiratory tract disease (URTD) [9, 10]. This syndrome, reported in both terrestrial and aquatic chelonian species, is characterized by serous, mucoid, or purulent nasal exudate, ocular and periorbital edema, excessive tearing, purulent ocular discharge, conjunctivitis, and lesions in the nasal epithelium and upper respiratory tract mucosa. In the most severe cases, it can lead to lethargy and death [9, 11].

*M. agassizii* was first identified and linked to URTD in desert [9] and gopher [10] tortoises in the United States. In the following years, *M. agassizii* infection was reported by serology, PCR, and/or culture in many species of free‐living and captive tortoises across the United States [12, 13, 14, 15, 16, 17, 18, 19, 20, 21], Mexico [22], Europe [23, 24, 25, 26, 27], and Africa [23, 28]. Interestingly, *M. agassizii* infection can manifest in both clinical and subclinical forms [6, 29]; moreover, convalescence/recrudescence cycles have been observed in both captive and free‐ranging tortoises [10, 30].

Despite its prevalence, the impact of *M. agassizii* infection on tortoises mortality, morbidity, and population viability remains poorly understood [6]. Specifically, the mechanisms of pathogenicity and the epidemiological relevance of related diseases are not fully clear. Furthermore, the contribution of distinct *M. agassizii* isolates to clinical manifestations and disease severity is ambiguous, largely due to a lack of effective tools for their identification and characterization [29]. To address this, Multilocus Sequence Typing (MLST) offers a powerful and practical approach for accurately comparing *Mycoplasma* isolates, providing essential insights into their genetic relationships and epidemiology. This method involves sequencing highly conserved housekeeping genes, enabling the discrimination of genetically similar strains. MLST has been widely applied to various *Mycoplasma* species, allowing for strain‐level differentiation and characterization [31, 32, 33, 34, 35, 36, 37, 38, 39, 40, 41, 42, 43, 44].

In this paper, we explore and propose a putative MLST scheme for *M. agassizii* typing, combining targeted PCR amplification with Nanopore sequencing. Ten housekeeping genes were selected and used to characterize *M. agassizii* strains from distinct tortoise populations in Italy and Portugal. The significance of this initial MLST method for identifying *M. agassizii* strains and its implications for understanding the epidemiological characteristics of URTD are also discussed.

## 2 Materials and Methods

### 2.1 Sampling and Selection of *M. agassizii* Positive Samples

A total of 127 choanal swabs were collected from 127 tortoises in 2010 and 2022−2023. Samples were collected from tortoises kept in wildlife recovery centers, zoological institutions, or under the care of private owners and breeders. Host species, sampling sites, and sampled individuals are reported in Table 1.

**TABLE 1:** Samples analyzed in this study.

Country	Sampling station	Species	No. of animals
Italy	Bonassai^a^	*Testudo graeca*	4
		*Testudo hermanni*	4
		*Testudo marginata*	16
	Monastir^a^	*Testudo graeca*	3
		*Testudo hermanni*	13
		*Testudo marginata*	9
			
Portugal	Alentejo^a^	*Centrochelys sulcata*	3
		*Stigmochelys pardalis*	1
	Algarve^a^	*Centrochelys sulcata*	7
		*Malacochersus tornieri*	3
		*Testudo graeca*	17
	Lisbon and Tagus Valley^b^	*Aldabrachelys gigantea*	3
		*Astrochelys radiata*	4
		*Centrochelys sulcata*	4
		*Geochelone elegans*	1
		*Stigmochelys pardalis*	6
		*Testudo hermanni*	4
		*Testudo horsfieldii*	4
		*Testudo marginata*	2
		*Testudo graeca*	9
	Northern Portugal^b^	*Chelonoidis carbonarius*	8
		*Indotestudo elongata*	2

^a^Wildlife Recovering Center or Zoo.

^b^Private owner or breeder.

Total DNA was extracted from the swabs using the QIAamp DNA Mini Kit (Qiagen) or Invisorb Spin Universal Kit (Invitek Molecular, Berlin, Germany) following the manufacturer's recommendations. Samples were screened for *M. agassizii* using previously described primers, Box‐F (5′‐CTGCTGTTATACAGAAAGAAAAG‐3′) and Box‐R (5′‐GACTTTGGGCATTACCGGC‐3′), which target a 996 bp region of the 16S‐23S rDNA intergenic region [24, 45]. Amplification was confirmed by 1% agarose gel electrophoresis, stained with GelRed (Biotium), and images were acquired with a Gel Doc EZ System (Bio‐Rad).

Twelve *M. agassizii* positive samples (six from Sardinia and six from Portugal) were selected for further molecular testing with *M. agassizii* housekeeping genes.

### 2.2 Selection of Housekeeping Genes for MLST and PCR Amplification

Ten candidate housekeeping genes were initially selected from previously published *Mycoplasma* MLST studies [31, 32, 33, 34, 35, 36, 37, 38, 39, 40, 41, 42, 43, 44, 46, 47]. Specific primers were then designed by aligning the sequences of these 10 genes of three *M. agassizii* strains available in GenBank: the type‐strain PS6^T^ (GCA_002272945), strain 723 (GCA_041379755), and 55P4 (GCA_041379755). Table 2 details the primer sequences, amplicon sizes, fragment positions within the PS6^T^ genome, and relative gene functions for each of the 10 tested housekeeping genes.

**TABLE 2:** Selection of candidate loci and primer design for *M. agassizii* MLST analysis.

Locus	Gene function	Primers	Position in the PS6^T^ genome	Selected for MLST
*uvrB*	Excinuclease ABC subunit B	Magassi_uvrB_270F TGACTACTATCGACCTGAAG Magassi_uvrB_1430R TGTTCACTATGGATGTAAGC	106444−108420	Yes
*tpiA*	Triosephosphate isomerase	Magassi_tpiA_43F ACTGCAAGCGAAGTTGAAAG Magassi_tpiA_707R CCTGCTACTAATGATGCTCC	110243−110983	Yes
*gyrB*	DNA gyrase subunit B	Magassi_gyrB_27F TTCAAGCAATATTCAGGTGC Magassi_gyrB_1271R TTGCTTGAACAATCTGCTAG	117665−119602	Yes
*fusA*	Elongation factor G	Magassi_fusA_118F CTAGGTGAAACTCATGATGG Magassi_fusA_1284R TAGGCGTTGTAGTCCAATTG	137033−139111	No
*efTu*	Elongation factor Tu	Magassi_Eftu_324F AACAGATGGACCTATGCCTC Magassi_Eftu_1010R GTACGGAAGTAGAATTGAGG	283001−284191	Yes
*rpoB*	DNA‐directed RNA polymerase subunit beta	Magassi_rpoB_26F GAGCATACCACTCGATTGAC Magassi_rpoB_1035R ATCACTAGTTGAGTGACGCC	283001−284191	Yes
*dnaA*	DnaA ATPase domain‐containing protein	Magassi_DnaA_72F GTCAGCTATTGATAATCAAC Magassi_DnaA_942R TCATGAAGGAATTGCTTCGC	308026−309408	No
*uvrA*	Excinuclease ABC subunit A	Magassi_uvrA_153F AGGTCGAAGAAGATATGTTG Magassi_uvrA_1228R TACAGTCAGCTTCTGACATG	389728−392553	Yes
*gmK*	Guanylate kinase	Magassi_gmK_21F AATCTTCGTTGGACCAAGTG Magassi_gmK_534R GGCATCATCATTAACAACTG	425486−426073	Yes
*atpG*	ATP synthase F1 subunit gamma	Magassi_atpG_30F ACGCAGTTTAGAGTTCTATC Magassi_atpG_993R AACTTTCTCAGCCACGAATG	564317−565156	Yes

All PCR amplifications were performed using Taq DNA Polymerase (Qiagen). Ten independent PCR reactions were set up with a final volume of 20 µL as follows: 1× CoralLoad PCR Buffer, 200 µM each dNTP, 0.8 µM each primer, 0.5 U Taq DNA Polymerase, and 2.5 µL of DNA template. The cycling conditions were: initial denaturation at 94°C for 3 min, followed by 35 cycles of 94°C for 1 min, 50°C for 1 min, and 72°C for 1 min, and a final elongation step at 72°C for 10 min. Amplicons were verified by agarose electrophoresis, as described above. Based on PCR results, eight candidate genes were selected for MLST analysis.

### 2.3 PCR Purification and Sequencing

PCR products were purified using the MinElute PCR Purification Kit (Qiagen) and quantified with a NanoDrop Lite Spectrophotometer (Thermo Fisher). Amplicon mixtures of the eight selected housekeeping genes were obtained by mixing equimolar quantities of PCR products from the same individual tortoise. These pooled amplicons were then subjected to Nanopore sequencing using the MinION Mk1C (Oxford Nanopore Technologies), following the manufacturer's recommendations. In order to run all samples on a single flow cell, the library was prepared by barcoding the 12 PCR product mixtures (representing the 12 individual tortoises) using the Native Barcoding Kit 24 V14 (Oxford Nanopore Technologies), according to its dedicated protocol. The library was then loaded and run on an ONT R10.4.1 flow cell. The sequencing run was set with the following parameters: 200 bp minimum read length; real‐time high‐accuracy basecalling; and a 16‐h runtime. Demultiplexed FASTQ files were merged, and reads were assembled and aligned to the reference genome using the Genome Detective platform (https://www.genomedetective.com).

### 2.4 Allele and Sequence Type Assignment

Sequences were initially analyzed using the DnaSP software [48]. The number of polymorphic sites, singletons, and alleles were computed for each locus.

Sequential numbers were assigned to all distinct alleles, and samples were subsequently classified into sequence types (STs) according to their allele combinations at the eight loci.

### 2.5 Data Analysis

The rate of nonsynonymous and synonymous substitutions (d_n_/d_s_) and the diversity index (H_d_) were calculated using the DnaSP software [48]. The discriminatory power of each MLST locus was determined using Simpson's index of diversity with 95% confidence intervals [49] with the Comparing Partitions online tool (http://www.comparingpartitions.info/).

The standardized index of association (I_a_) was calculated using the LIAN (LInkage ANalysis) tool (http://guanine.evolbio.mpg.de/cgi‐bin/lian/lian.cgi.pl), applying the Monte Carlo test (100 iterations) [50]. To define the discriminative power of a minimal set of loci, LIAN analysis was also performed on loci sets defined by three different H_d_ cut‐offs (≥ 0.7, ≥ 0.8, ≥ 0.83, and ≥ 0.85).

The ST dendrogram was generated using the SRplot online tool (https://www.bioinformatics.com.cn/SRplot) [51].

### 2.6 Phylogenetic Analysis

For each ST, the eight loci sequences were concatenated and aligned with MEGA11 [52]. Phylogenetic trees were generated using the Maximum Parsimony (MP), Minimum Evolution (ME), and Maximum Likelihood (ML) methods. All trees were bootstrapped with 1000 replicates [53]. MP trees were obtained using the Subtree‐Pruning‐Regrafting (SPR) algorithm [54] (search level 1), where initial trees were generated by the random addition of sequences (10 replicates). For ML trees, evolutionary distances were computed using the Maximum Composite Likelihood method [55] and the GTR model (identified in MEGA as the most fitting model). ME trees were based on the Close‐Neighbor‐Interchange (CNI) algorithm [54] (search level 1). The Neighbor‐Joining (NJ) algorithm [56] was used to generate the initial tree. In all phylogenetic analyses, concatenated *Mycoplasma pulmonis* (*M. pulmonis*) sequences were used as outgroup.

## 3 Results

### 3.1 Identification of *M. agassizii* Positive Samples

A total of 73/127 (57.5%) samples tested positive for *M. agassizii* by Box PCR (Table 3). Sanger sequencing confirmed all amplicons identity as *M. agassizii*.

**TABLE 3:** Details on the Box PCR results performed on tortoise swab samples.

Sampling site	No. of samples	No. of positive samples	% of positive samples
Italy	49	22	44.9%
Portugal	78	52	66.7%
**Total**	**127**	**73**	**57.5** **%**

Total values (sums) are highlighted in bold.

### 3.2 Housekeeping Gene Amplification and Sequence Variation Analysis

**FIGURE 1:**
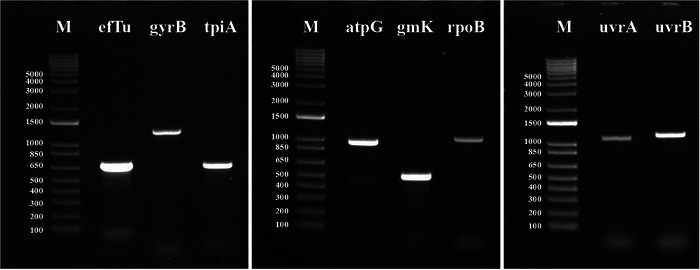
Representative image of housekeeping gene PCRs included in this study. M = 1 kb plus DNA ladder (Invitrogen).

All candidate housekeeping genes, except *fusA* and *dnaA* loci, were successfully amplified from all samples. Therefore, only eight genes (*uvrB*, *tpiA*, *gyrB*, *efTu*, *rpoB*, *uvrA*, *gmK*, and *atpG*) were considered for the development of an *M. agassizii* MLST potential scheme (Figure 1). Amplicon sizes consistently matched their in silico estimated lengths. Individual amplicon mixtures were efficiently barcoded and sequenced in a single Nanopore sequencing session. All sequences obtained via Nanopore sequencing were further validated by traditional Sanger sequencing. Sequences were deposited in GenBank under accession numbers PV614338−PV614349 and PV642936−PV642995.

All eight *M. agassizii* genes analyzed in this study showed genetic variability. Table 4 summarizes the sequence variation parameters for each locus. Briefly, the highest genetic variation was detected in *uvrB*, with 84 polymorphic sites leading to 11 different alleles, while the *uvrA* locus contained 75 polymorphic sites but yielded only six alleles. The haplotype diversity (H_d_) for each locus ranged from 0.714 (*uvrA*) to 0.952 (*uvrB*). Similarly, Simpson's index of diversity spanned from 0.681 (*uvrA*) to 0.956 (*uvrB*). The standardized index of association (I_a_) calculated by LIAN was 0.5846.

**TABLE 4:** Details on the analysis of the eight loci used in the *M. agassizii* MLST scheme.

**Locus**	**Analyzed sequence (bp)**	**No. of polymorphic sites**	**No. of singletons**	**No. of alleles**	**H_d_**	**d_n_/d_s_**	**Simpson's ID**
*uvrB*	1121	84	46	11	0.952	0.048	0.956
*tpiA*	625	51	38	7	0.724	0.102	0.692
*gyrB*	1205	86	30	8	0.838	0.034	0.824
*efTu*	647	11	8	7	0.829	*	0.813
*rpoB*	970	57	36	10	0.924	*	0.923
*uvrA*	1036	75	48	6	0.714	0.114	0.681
*gmK*	474	28	18	8	0.838	0.526	0.824
*atpG*	924	38	24	7	0.724	0.05	0.692

Abbreviations: dn/ds, ratio of nonsynonymous to synonymous mutations; Hd, genetic diversity; Simpson's ID, Simpson's index of diversity.

* Only synonymous mutations were detected.

### 3.3 Determination of Allelic Profiles and Sequence Types

Sequences analysis allowed discriminating different allelic versions for each locus, and their combinations were used to define STs. Haplotypes and ST definitions are reported in Table 5. In total, 14 STs were discriminated and defined among the 15 different isolates, which included one type strain, two GenBank field isolates, six Italian isolates, and six Portuguese isolates. The three genomes deposited in GenBank generated three different STs (ST1−ST3). Among the Sardinian samples, five different STs were identified (ST4−ST8). Portuguese isolates generated six different STs (ST9−ST14) with allelic overlaps observed across different loci.

**TABLE 5:** Description of strains used in this study, their ST, and allelic profiles.

Isolate	Host ID	Host species	ST	Sampling location	*uvrB*	*tpiA*	*gyrB*	*efTu*	*rpoB*	*uvrA*	*gmK*	*atpG*
PS6^T^ (ATCC 700616)	GaS6	*Gopherus agassizii*	1	Nevada (USA)	1	1	1	1	1	1	1	1
723 (ATCC 700617)	Gp723	*Gopherus polyphemus*	2	Florida (USA)	2	2	2	2	2	2	2	2
55P4	Tk55P4	*Testudo kleinmanni*	3	Austria	3	3	3	3	3	3	3	3
Sar1	TmN	*Testudo marginata*	4	Bonassai (Italy)	4	4	4	4	4	4	4	4
Sar1	ThSCH	*Testudo hermanni*	4	Bonassai (Italy)	4	4	4	4	4	4	4	4
Sar2	Tm20	*Testudo marginata*	5	Bonassai (Italy)	5	5	5	5	5	5	5	5
Sar3	Th7	*Testudo hermanni*	6	Monastir (Italy)	6	6	6	6	6	6	6	6
Sar4	Tm8	*Testudo marginata*	7	Monastir (Italy)	7	6	6	7	7	6	7	6
Sar5	Tg9	*Testudo graeca*	8	Monastir (Italy)	8	6	7	7	8	6	7	6
PT1	SpB4	*Stigmochelys pardalis*	9	Alentejo (Portugal)	9	7	8	5	9	5	8	7
PT2	ArD3	*Astrochelys radiata*	10	Lisbon and Tagus Valley (Portugal)	10	6	6	6	7	6	6	6
PT3	CcG12	*Chelonoidis carbonarius*	11	Northern Portugal	7	6	6	7	10	6	7	6
PT4	TgH1	*Testudo graeca*	12	Algarve (Portugal)	11	6	6	7	7	6	7	6
PT5	GeI1	*Geochelone elegans*	13	Lisbon and Tagus Valley (Portugal)	10	6	6	7	1	6	7	6
PT6	TgJ11	*Testudo graeca*	14	Algarve (Portugal)	10	6	7	7	7	6	7	6

Abbreviation: ST, sequence type.

The minimal set of loci required to discriminate the different STs was tentatively calculated by applying different H_d_ cut‐offs. As presented in Table 6, increasing the H_d_ cut‐off to 0.83 still allowed the discrimination of all STs based on four loci (*uvrB*, *gyrB*, *rpoB*, and *gmK*). This trend was accompanied by a decrease in linkage disequilibrium, with a value of 0.2246 at H_d_ ≥ 0.83 cut‐off.

**TABLE 6:** Discriminating power of loci sets at different H_d_ cut‐offs.

H_d_ cut‐off	No. of loci	No. of STs	I_a_
≥0.7	8	14	0.5846
≥0.8	5	14	0.3330
≥0.83	4	14	0.2246
≥0.85	2	13	0.1472

**FIGURE 2:**
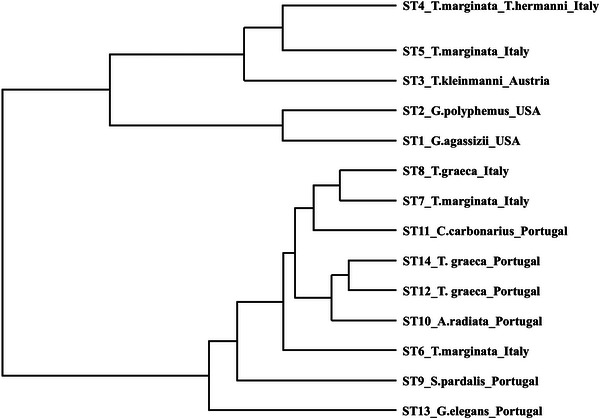
Dendrogram illustrating the relationships between STs found in studied samples.

The ST dendrogram splits STs into two main groups (STs 1−5 and STs 6−14). However, these groups did not support any clear geographical or host‐specific stratification (Figure 2).

### 3.4 Phylogenetic Analysis

**FIGURE 3:**
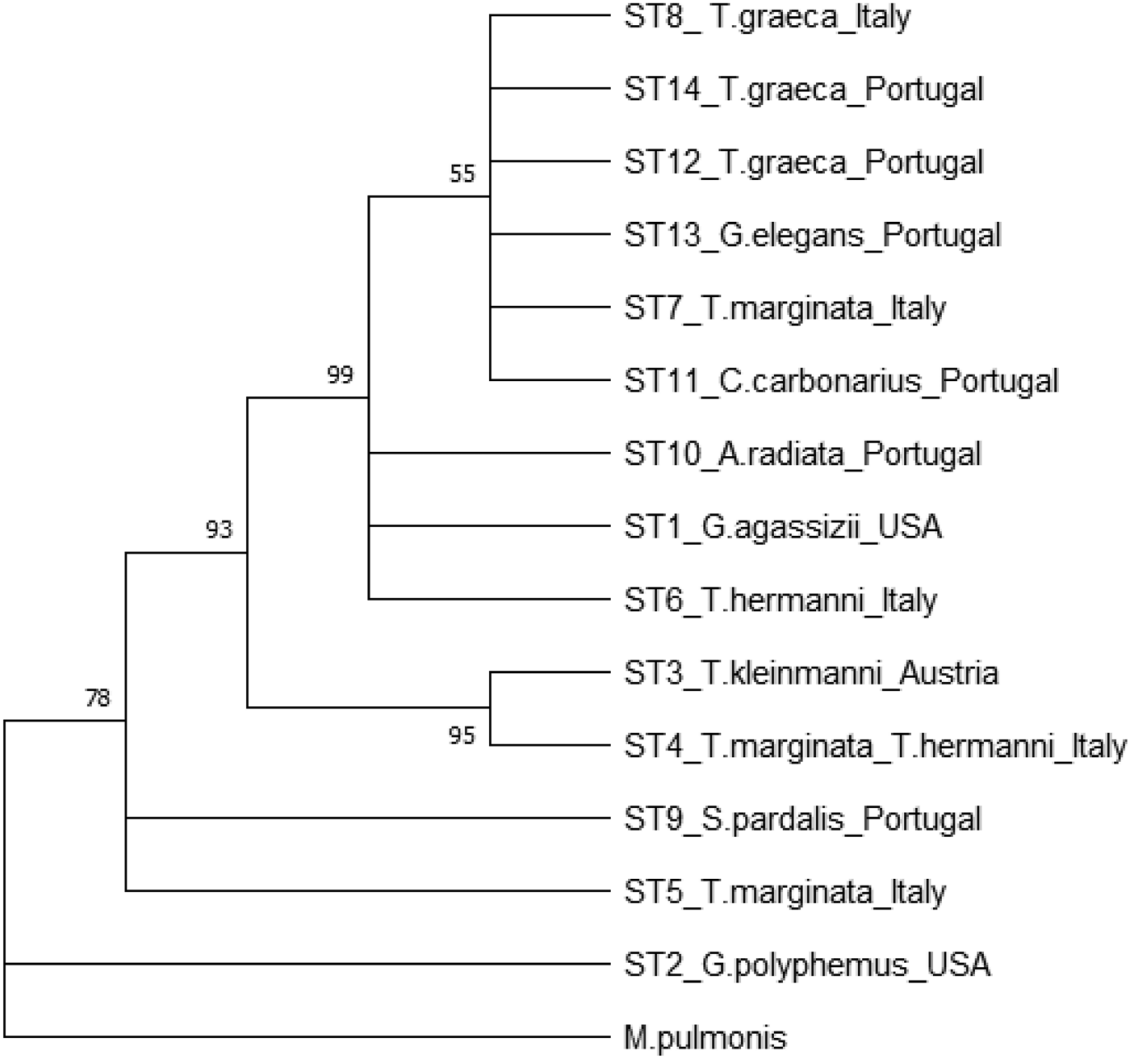
Representative phylogenetic tree. The percentage of replicate trees in which the associated taxa clustered together in the bootstrap test (1000 replicates) are shown next to the branches. Branches corresponding to partitions reproduced in less than 50% bootstrap replicates were collapsed.

Sequences obtained by MLST PCRs were concatenated and used for phylogenetic analysis. The trees generated by the MP, ME, and NJ methods were congruent (Figure 3). Consistent with the dendrogram analysis, the phylogenetic analyses did not support any stratification of *M. agassizii* based on host species or sampling geographic locations.

## 4 Discussion

*M. agassizii* is a significant worldwide pathogen of terrestrial tortoises. To mitigate the harmful effects of *M. agassizii* on tortoise species, it is crucial to monitor and control its spread among different populations. The clinical outcome of *M. agassizii* infection is multifactorial, influenced by both host and pathogen factors, ranging from subclinical cases to the development of URTD. Understanding the contribution of distinct strains to disease development depends on the ability to identify genetically distinct strains and correlate them with specific host conditions. To date, genetic characterization and comparison of *M. agassizii* isolates have been limited. While some studies have reported limited genetic variation among field isolates collected from Mojave and Sonoran Desert tortoises, these findings are constrained by methodological limitations, including the small number of isolates analyzed and the fact that all samples originated from asymptomatic individuals. Furthermore, previous studies have primarily targeted the 16S rRNA gene, which, although suitable for species‐level identification, lacks the discriminatory power necessary to resolve intraspecific variation among isolates.

This study presents the first proposed set of genetic loci for the development of an MLST scheme for *M. agassizii*. An initial panel of 10 housekeeping genes was evaluated using 12 selected field samples (six from Italy and six from Portugal). Two loci were excluded due to inconsistent PCR amplification, with subsequent analyses focusing on the remaining eight loci that consistently amplified across all samples. The proposed MLST scheme was also applied in silico to isolates PS6^T^, 723, and 55P4, for which whole‐genome sequences are publicly available in GenBank. Based on these eight loci, the 15 isolates were sorted into 14 distinct STs. Unexpectedly, high genetic diversity among *M. agassizii* isolates was detected even in closely spaced and overlapping sampling locations. Only one ST (ST4) was found in more than one individual, occurring in two tortoises housed at the same Italian rescue center. It is plausible that at least one of these turtles contracted the infection within the rescue center. Notably, a third sample from the same rescue center showed a completely different allelic profile across all eight loci and was ascribed to ST5. Three distinct STs (ST6−ST8) were identified in a second Italian rescue center, with some overlapping alleles. Interestingly, there was no allelic overlap between the STs identified in the two Italian rescue centers or with the GenBank strains (PS6^T^, 723, and 55P4).

Portuguese isolates exhibited greater overlap among different STs and shared numerous allelic variants with the Sardinian samples. Additionally, they introduced novel alleles at all analyzed loci, except for *efTu* and *uvrA*. Notably, ST13 shares the *rpoB* allele with the US 723 strain (ST2). Preliminary cluster and phylogenetic analyses indicate that the different STs do not segregate by geographical origin or tortoise species. This pattern may result from several factors, including mixed‐species housing in rescue centers and enclosures, suboptimal hygienic conditions, insufficient health screening upon admission, and even illegal trade of exotic species. However, further speculation is limited by the current small sample size.

To investigate the minimal number of loci necessary to discriminate all STs using MLST, we progressively applied different H_d_ cut‐offs and recorded the number of STs recognized at each cut‐off, alongside the corresponding number of required loci. Four loci (H_d_ ≥ 0.83) were sufficient to differentiate the 14 STs, yielding an I_a_ value of 0.2246. An I_a_ value of 0.22 in the MLST analysis of *M. agassizii* indicates a moderate level of linkage disequilibrium among the analyzed loci. This suggests that the population structure of *M. agassizii* is partially clonal, with evidence of genetic recombination events occurring, though not at a frequency sufficient to fully randomize allele associations. Such a pattern is consistent with a bacterial species that primarily undergoes clonal reproduction but retains some capacity for horizontal gene transfer or recombination. In the context of *M. agassizii*, this intermediate I_a_ value reflects a balance between vertical inheritance and genetic exchange, which may influence strain diversity and adaptation within tortoise populations. These findings support the utility of the selected MLST loci for strain differentiation while providing insights into the evolutionary dynamics of *M. agassizii* populations. Nevertheless, given the limited number of isolates analyzed in this study, we recommend testing a greater number of strains with all eight proposed loci. Only after a broader analysis can a minimal set of genes for a reliable *M. agassizii* MLST be definitively established. Moreover, greater effort should be invested in sampling and testing tortoises exhibiting signs of URTD.

## 5 Conclusion

The development of a reliable MLST scheme for *M. agassizii* with adequate discriminatory power is crucial for providing deeper insights into its epidemiology, pathogenicity, and distribution. Such a tool would also facilitate the implementation of effective URTD control programs. To date, there is no suitable method for comparing and characterizing *M. agassizii* isolates or for associating specific STs with pathogenic traits and clinical outcomes. A specific strain identification method could reduce the need for culling infected tortoises, thereby benefiting host conservation efforts. Furthermore, pathogen typing would enable the tracking of illegal trade of these animals. In conclusion, the proposed MLST method should be applied to a larger collection of bacterial isolates, including those obtained from symptomatic animals, to clarify the contribution of specific *M. agassizii* strains to URTD in terrestrial tortoises.

## Author Contributions

A.A. and C.C. designed the study; C.C., A.A., R.L., M.L., and R.P. collected the samples; C.C., R.Z., M.B., M.L., A.V., and M.A. performed the experiments; A.A. and C.C. analyzed the data; A.A. and C.C. wrote the manuscript; M.P., A.V., R.L., and M.A. revised the manuscript.

## Conflicts of Interest

The authors declare no competing or financial interests.
